# Assessing the International Spreading Risk Associated with the 2014 West African Ebola Outbreak

**DOI:** 10.1371/currents.outbreaks.cd818f63d40e24aef769dda7df9e0da5

**Published:** 2014-09-02

**Authors:** Marcelo F. C. Gomes, Ana Pastore y Piontti, Luca Rossi, Dennis Chao, Ira Longini, M. Elizabeth Halloran, Alessandro Vespignani

**Affiliations:** Laboratory for the Modeling of Biological and Socio-technical Systems, Northeastern University, Boston, Massachusetts, USA; Laboratory for the Modeling of Biological and Socio-technical Systems, Northeastern University, Boston, Massachusetts, USA; Institute for Scientific Interchange (ISI), Torino, Italy; Vaccine and Infectious Disease Division, Fred Hutchinson Cancer Research Center, Seattle, Washington, USA; Department of Biostatistics, University of Florida, Gainesville, Florida, USA; Vaccine and Infectious Disease Division, Fred Hutchinson Cancer Research Center and Biostatistics, University of Washington, Seattle, Washington, USA; Laboratory for the Modeling of Biological and Socio-Technical Systems, Northeastern University, Boston, Massachusetts, USA

**Keywords:** 2014WA, disease model, disease outbreak, EVD, infectious disease

## Abstract

Background: The 2014 West African Ebola Outbreak is so far the largest and deadliest recorded in history. The affected countries, Sierra Leone, Guinea, Liberia, and Nigeria, have been struggling to contain and to mitigate the outbreak. The ongoing rise in confirmed and suspected cases, 2615 as of 20 August 2014, is considered to increase the risk of international dissemination, especially because the epidemic is now affecting cities with major commercial airports. Method: We use the Global Epidemic and Mobility Model to generate stochastic, individual based simulations of epidemic spread worldwide, yielding, among other measures, the incidence and seeding events at a daily resolution for 3,362 subpopulations in 220 countries. The mobility model integrates daily airline passenger traffic worldwide and the disease model includes the community, hospital, and burial transmission dynamic. We use a multimodel inference approach calibrated on data from 6 July to the date of 9 August 2014. The estimates obtained were used to generate a 3-month ensemble forecast that provides quantitative estimates of the local transmission of Ebola virus disease in West Africa and the probability of international spread if the containment measures are not successful at curtailing the outbreak. Results: We model the short-term growth rate of the disease in the affected West African countries and estimate the basic reproductive number to be in the range 1.5 − 2.0 (interval at the 1/10 relative likelihood). We simulated the international spreading of the outbreak and provide the estimate for the probability of Ebola virus disease case importation in countries across the world. Results indicate that the short-term (3 and 6 weeks) probability of international spread outside the African region is small, but not negligible. The extension of the outbreak is more likely occurring in African countries, increasing the risk of international dissemination on a longer time scale.

## Introduction

The outbreak of Ebola virus disease (EVD) that started in December 2013 has defied several months of mitigation and containment efforts. In July 2014 it was still evolving in Guinea, Liberia and Sierra Leone. As of 20 August, the toll in those countries had reached 844 EVD confirmed deaths 1. On 20 July, the outbreak reached Nigeria through an infected traveler coming from Liberia. The Nigerian official reports list 12 probable cases, and it is not clear if the outbreak has been contained.

EVD is caused by infection with a virus of the family Filoviridae, genus Ebolavirus 2. EVD transmission during the incubation period is very unlikely and occurs via direct contact with blood, secretions, and/or other bodily fluids of dead or living infected persons. Gene sequencing of the virus causing the 2014 West African (2014WA) outbreak has demonstrated 98% homology with the Zaire Ebola virus, with a 55% case fatality ratio (CFR) across the affected countries 3. Unfortunately there are no licensed treatments available for EVD, and severely ill patients can only be cared for with intensive supportive care.

The 2014WA outbreak is the largest ever observed, both by number of cases and geographical extension. For this reason, on 6-7 August, an Emergency Committee of the WHO 4 advised the 2014WA outbreak constitutes an ’extraordinary event’ and a public health risk to other States. Indeed, although the outbreak started in an isolated region of Guinea, transmission has occurred in large cities (Conakry, Freetown, Monrovia and Lagos) of the four affected countries. These urban areas have major international airports, thus raising concern about a quick internationalization of the outbreak (see Fig. 1). While importation of cases should not generate large outbreaks in countries where prompt isolation of cases in appropriate health care facilities occurs, it is clear that a quantitative analysis of the risk of importation of cases (likelihood, timeline, number of cases) in countries not affected at the moment by the outbreak may provide valuable intelligence on the evolution of the 2014WA outbreak.

So far most of the analyses on the risk of international spread of the outbreak have focused on the analysis of the sheer volume of international passenger traffic across countries 5 ^,^ 6. These analyses however do not consider the local evolution of the outbreak in the affected countries and the specific etiology of the disease (incubation time scale, etc.). Here we provide a quantitative assessment of the international spread based on large-scale computer microsimulations of the 2014WA outbreak that generate stochastic simulations of epidemic spread worldwide, yielding, among other measures, the case importation events at a daily resolution for 3,362 subpopulations in 220 countries. We use the Global Epidemic and Mobility Model that integrates high-resolution data on human demography and mobility on a worldwide scale in a metapopulation stochastic epidemic model 7 ^,^ 8 ^,^ 9. The disease dynamics within each population consider explicitly that EVD transmissions occur in the general community, in hospital settings, and during funeral rites 10. For parameter inference, we use a Monte Carlo likelihood analysis that considers more than 1,000,000 simulations that sample the disease model space and the data on the 2014WA outbreak up to 9 August 2014. This approach selects the disease dynamic model that we use to generate numerical stochastic simulations of an epidemic’s local (within West African countries) and global progression.

We evaluate the progression of the epidemic in West Africa and its international spread under the assumption that the EVD outbreak continues to evolve at the current pace. The numerical simulation results show a steep increase of cases in the West Africa region, unless the transmissibility of the EVD is successfully mitigated. The overall basic reproductive number of the epidemic in the region is estimated to be in the range 1*.*5 *− *2*.*0. We find that, although surveillance and containment measures have been in place for several months, the transmissibility in hospital and funeral rites are likely an appreciable component of the overall transmissibility. The probability of case exportation is extremely modest (upper bound less than 5%) for non-African countries, with the exception of the United Kingdom (UK), Belgium, France and the United States (US). As of the beginning of September, the countries with the largest probability of seeing the arrival of EVD cases are Ghana, UK and Gambia. The overall probability of international spread will increase if the Nigerian outbreak is not promptly controlled. We also show that as of the end of September, the size distribution of outbreaks due to the international spread of the EVD is contained (median value *<4* cases) for countries outside of the African region. Severe travel restrictions to and from the affected areas (80% airline traffic reduction) generates only a 3-4 weeks delay in the international spreading.

The lack of detailed data on the 2014WA EVD outbreak makes any modeling approach vulnerable to the many assumptions and uncertainty about basic parameters and the quality of data. However, we hope that the characterization of the EVD 2014WA outbreak and the associated risk of international spread provided here may be useful to national and international agencies in allocating resources for interventions to contain and to mitigate the epidemic.

**Air traffic connections from West African countries to the rest of the world:**
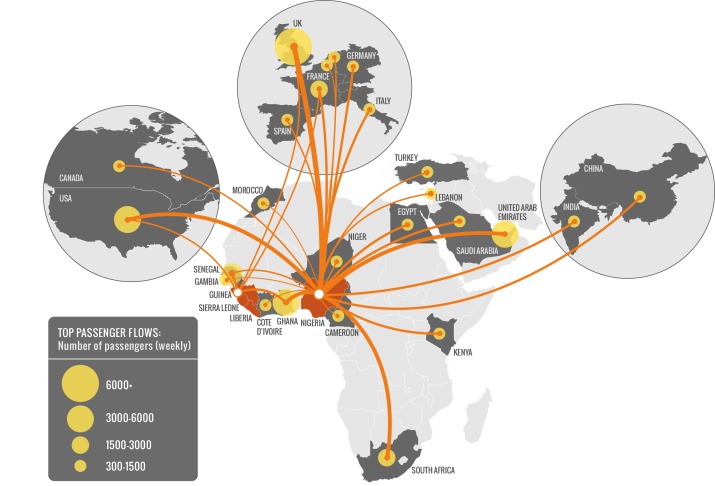
Air traffic connections from West African countries to the rest of the world. Guinea, Liberia, and Sierra Leone are not well connected outside the region. Nigeria, in contrast, being the most populous country in West Africa with more than 166 million people, is well connected to the rest of world. For historical reasons, all these countries have the strongest ties with European countries.

## Results

To provide a quantitative analysis of the risk of international spread of the EVD 2014WA outbreak, we use a data-driven global stochastic and spatial epidemic model 7 ^,^ 8 ^,^ 9. Details of the models are reported in the Methods section. The model generates microsimulations at the individual level that provide a stochastic ensemble of possible epidemic evolutions for each identical set of initial conditions and disease parameters. These simulations can be used to provide statistical estimates as newly generated cases, importation events, and time of arrival of the infection whose values depend on the key disease parameters determined by the calibration of the model. The adopted EVD modeling scheme 10 includes hospitalized and funeral compartments. To further support the obtained results we also considered a parsimonious susceptible, exposed, infectious and recovered (SEIR) disease scheme 11. We have considered the transmissibility component as the key parameter to be determined from data. We have also considered the current CFR (55%) from the 2014WA outbreak data 1. The remaining parameters (reported in the Methods section) follow from the study of Legrand *et al.* 10 and are consistent across the modeling literature for different outbreaks 11 ^,^ 12.

**Cumulative number of EVD deaths in West Africa as of 1 July 2014:**
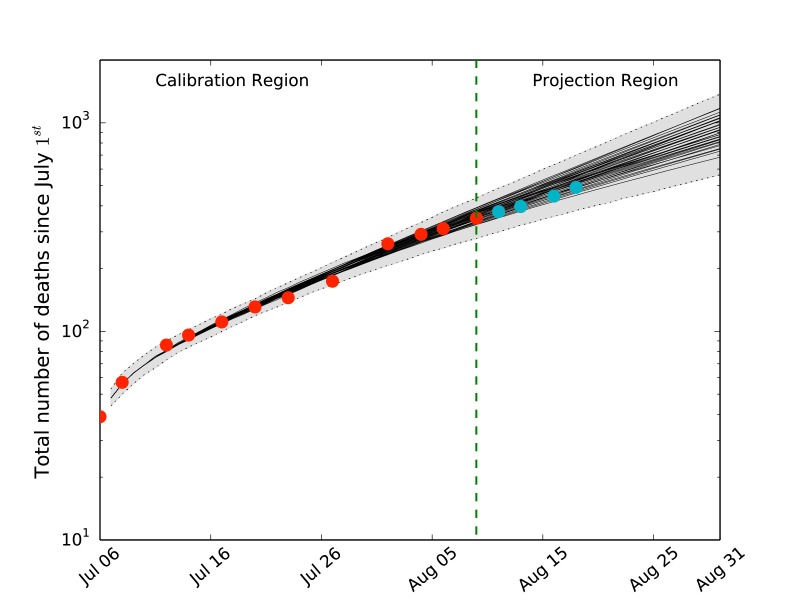
Cumulative number of EVD deaths in Sierra Leone, Guinea and Liberia as of 1 July 2014. The dots correspond to the data from the official WHO reports. The red dots were used for the model calibration. The blue dots are experimental data points received after the calibration of the model and are reported for the purpose of comparing with the model projections. The black thin lines are the expected values for the models selected by the likelihood analysis. The grey areas correspond to the 95% reference range provided by the fluctuations of the stochastic microsimulations. The green line divides the WHO data region used for the model selection from the projection region.

**Risk of EVD case importation:**
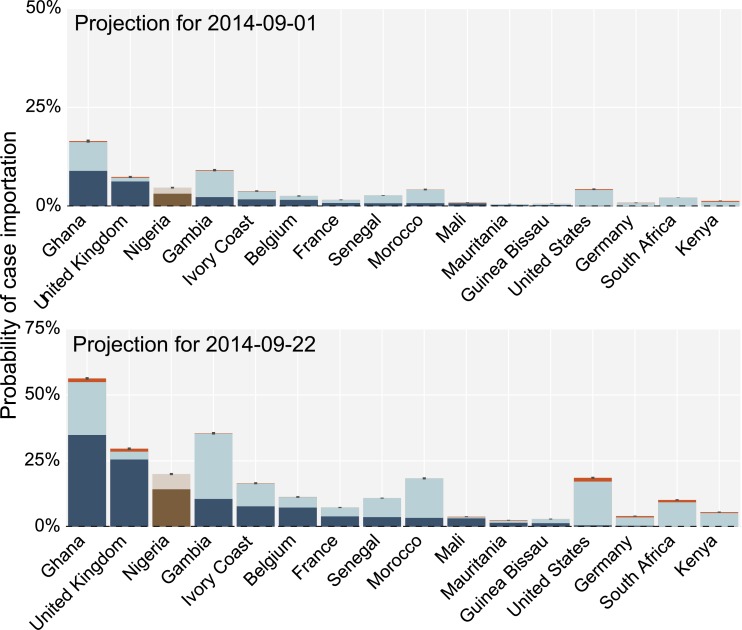
Top 16 countries at risk of EVD case importation in the short term: (top) 1 September and (bottom) 22 September 2014. The risk is assessed as the probability that a country will experience at least one case importation by the corresponding date, conditional on not having imported cases prior to 21 August 2014. The dark blue and light blue bars represent the minimum and maximum probability estimates, respectively, according to different models of case detection during travel (see text). The orange area corresponds to the probability maximum assuming the Nigerian outbreak starts to follow the same dynamic of the other West African countries affected by the EVD epidemic. We report the rank of Nigeria as well, which has experienced already a case importation on 20 of July and indeed it ranks among the countries with the larger probability of case importation.

**EVD outbreak size distribution:**
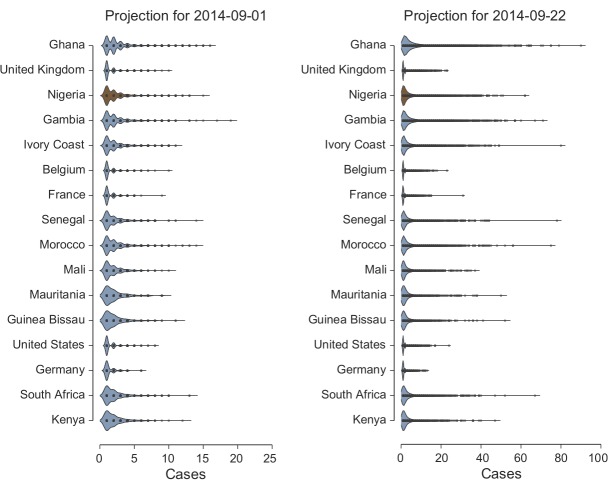
Kernel density plots reproducing the distribution of the EVD outbreak size in countries that experience case importation at two different dates: (left) 1 September, (right) 22 September, conditional on not having imported cases prior to 21 August 2014. The outbreak size considers the imported case(s) and the local transmission events. The distribution is obtained by analyzing 10,000 microsimulations of the models selected by the relative likelihood analysis. The dots inside the violin plots represent distribution points. The same plots for the case in which the outbreak in Nigeria is not contained show small variations that do not alter the overall picture.

**Local transmission and the EVD dynamic in West Africa**

To estimate the key parameters, we consider data after 7 July 2014. As of that date, the outbreak had already been unfolding for several months and we can consider that interventions to reduce transmission were already in place, and thus included in the effective value of the transmissibility. By using the approach detailed in the Methods section for the EVD model including hospital and funeral settings 10, we estimate an overall basic reproductive number *R*_0_ = 1*.*8 [1*.*5 *− *2*.*0] (interval at the 1/10 relative likelihood). The parsimonious SEIR model estimates *R*_0_ = 2*.*1 [1*.*9 *− *2*.*4], although its relative likelihood is less than 1/100. In the Legrand et al. model 10 the basic reproductive number can be written as the sum of three terms relative to the transmission in the community, hospital and funeral settings, yielding *R*_*I *_= 0*.*8 [0*.*3 *− *0*.*9], *R*_*H *_= 0*.*4 [0*.*2 *− *1*.*4] and *R*_*F *_= 0*.*6 [0*.*2 *− *1*.*0], respectively. The results suggest that although containment measures have been in place for several months, the likelihood of appreciable transmission in all settings is relevant. It must be noted however that the transmissibility in the various settings is difficult to determine as different partitions of the transmissibility can provide similar growth rates for the epidemic, and the available data may not be enough to discriminate otherwise. In Fig. 2 we plot the average behavior of the selected models compared to the WHO official data. The new data collected from the WHO after 9 August are within the 95% reference range of the projected behavior. Although this hints that the model is capturing the EVD dynamic in the West African regions, it also indicates that surveillance and containment measures are not yet sufficiently lowering the transmission rate to push the number of observed cases below the projected value.

**International spread**

The microsimulations generated by the selected models allow tracking the importation of EVD cases to other countries of the world from the West African countries affected by the outbreak. Concurrently to modeling the evolution of the EVD in the affected countries, our computational approach simulates the number of passengers traveling daily worldwide on each airline connection in the world. To quantify the risk of international spread, we consider the models selected with the multimodel selection approach to best represent the local EVD dynamic in West Africa and perform for each model 1,000 microsimulations. In each microsimulation, we monitor the arrival of EVD exposed individuals in countries across the world at a daily scale and estimate the probability of each country being invaded on 1 and 22 September 2014. These probabilities are conditional on the fact that no countries had confirmed EVD importation until 21 August 2014. We have also taken into account that Ivory Coast, Senegal, Guinea and Mauritania have closed their borders with the EVD affected area. Concerning Nigeria, we consider two scenarios: the first one in which the outbreak is contained, and one in which outbreak follows the same dynamic of EVD in Sierra Leone, Liberia, and Guinea. Furthermore, in assessing case importation by airline travel it is also important to consider if the EVD cases are detected during a connecting flight or at the final destination of the traveler. In our analysis we have implemented two different models: i) the EVD cases are identified after the first connecting flight; ii) the EVD cases are able to travel to their final destination. These models provide a minimum and maximum for the probability of case importation in each country, whose spread depends on whether the country's transportation systems act as a traffic gateway or a destination hub.

In Fig. 3 we report the top 15 countries plus Nigeria ranked according to their probability of case importation, conditional on the fact that they have not seen imported cases before August 21 (probability of case importation for countries not reported in the figure is available upon request to the authors). We also show the risk of importation for Nigeria. Although Nigeria has already received one case importation, it is relevant in supporting the obtained results to observe that it ranks as one of the countries with the largest probability of importation. It is possible to observe that the probability of importation is relatively small (*<5*%) for countries not in the African region, with the exception of the United Kingdom (UK). The probability of importation in a four weeks horizon (22 September) increases, but does not exceed the 25% threshold with the exception of Ghana and Gambia. It is important to stress that since we are talking of cumulative probabilities, they shall be constantly recalculated conditional on the fact that the importation has not been observed up to a given date. The risk of importation increases if the Nigerian outbreak is not contained. However the increase is more notable for the 22 September projection, as the Nigerian outbreak is very recent and needs time before it can add a critical mass of EVD cases to the spreading dynamics. In the results of Fig. 3 we do not take into account that some airlines have already decided to interrupt connections to the EVD-affected region. This analysis has to be considered as a baseline case where no traveling interventions are considered. For this reason we performed a sensitivity analysis in which we considered an 80% airline traffic reduction from and to the West African countries affected by the outbreak. The results obtained show a considerable reduction of the probability of case importation. However the probability of importation increases with time, and the net effect is essentially a mere three-week shift in the time progression for the probability of case importation (results not shown).

A further quantity we can monitor is the number of imported and locally generated cases we can expect in each country conditional on the event of case importation. When an EVD carrier is arriving in a country not affected by the outbreak, we look at the local transmission and measure the size of the epidemic cluster generated by the index case. We assume that in countries outside Africa, the hospital and funeral transmissibility is null. This assumption, perhaps optimistic, is motivated by the current worldwide awareness of the possibility of EVD cases and the alert of public health agencies worldwide. Furthermore at this stage, we are interested in the risk of international spread from the West Africa region, and it is too early to make any inference about transmissibility of the disease in other socio-cultural settings. In Fig. 4 we show the kernel density plots of the distribution of the outbreak size, conditional on the occurrence of importation of EVD cases, for the top 16 countries at risk of importation (Fig. 3). We observe that the expected value of the cluster size in the case of international spread is always rather small (in all countries mean<6; median<4). Large outbreak involving more than 10 individuals although potentially possible can be considered as very rare events (Detailed statistics per country are available upon request). This numerical evidence is good news, as it points out that effective management and isolation of cases is keeping the number of EVD cases to deal with to a very limited number, lowering the risk of losing control of the outbreak.

## Discussion

The 2014WA EVD outbreak is progressing at a fast pace and is considered an international health threat. We provide a quantitative analysis of the potential international spread that may inform policy making and the discussion concerning risk in countries outside the West Africa region.

Sustained local transmission of the EVD is currently observed in Sierra Leone, Liberia, and Guinea. Nigeria has experienced local transmission from a carrier traveling from Liberia, and it is not clear as of 21 August if the outbreak is contained or not. The estimates of the transmissibility indicate a reproductive number *R*_0_ in West Africa ranging from 1*.*5 to 2*.*0. The transmissibility found here is in the range of estimates in previous outbreaks, however it considers that we are analyzing the outbreak starting on the date of 6 July, when intervention measures have been implemented for several months. Unfortunately the modeled growth rate of the epidemic indicates that more aggressive implementation of the surveillance and containment policies have to be adopted. It is important to stress that these estimates are obtained by assuming other parameters of the 2014WA outbreak (incubation time, time from onset to hospitalization, etc.) to be in the range of estimates from past outbreaks 10 . More data and further research is needed to provide independent estimates specific for the 2014WA outbreak. Another confounding factor in obtaining the provided estimates for the transmissibility is the likely underreporting of cases and deaths in the affected countries. We have provided a sensitivity analysis that assumes a 50% underreporting in the data about hospitalizations and deaths. In this case, the transmissibility estimate extends the range of allowed values up to *R*_0_ = 2*.*5. Although this analysis does not drastically alter the picture offered by the baseline analysis, even more severe underreporting, possibly heterogeneously distributed, could be explored. Finally, estimates are obtained assuming the same EVD dynamic and transmissibility in all affected countries. A refined analysis should consider different EVD dynamics in Sierra Leone, Guinea and Liberia by performing analyses on the respective datasets. This analysis however leads to a high dimensional parameter space that requires further numerical and analysis work.

The probability of any country to experience EVD case importation depends on the passenger flow from the areas affected by the outbreak, the case numbers and the duration of the incubation time. To characterize and forecast the international spread, we assume that the transmissibility of the EVD, i.e. the growth rate of the epidemic, in the affected regions is not changing in the next two months. The number of cases generated in the affected countries is therefore supposed to follow the behavior reported in Fig. 2. However, the transmissibility may change as the epidemic progresses. For instance, new containment measures will lower the local transmissibility with a decrease of observed cases. While this is the scenario we hope will occur in the future, if the containment measures are not successful we may expect a deterioration of the health infrastructure capabilities in the EVD-affected areas. This would likely lead to the increase in transmissibility and an acceleration of the epidemic. For this reason the projections offered for the international spread can be only considered as a baseline scenario. The evolution of the epidemic is likely to deviate from the current behavior in the next few weeks/months and the model calibration and selection, and the ensuing analysis should be constantly updated as new data and evidence are gathered from the affected regions. Further and more detailed explorations of the possible effectiveness of isolation and containment measures requires much more detailed data about transmission chains, including time of onset of symptoms, who the contacts are, and whether they get infected. Information about variation in the mobility patterns of people with the neighboring countries is also crucial.

The short-term projection for the international spread of the EVD shows a small probability for countries outside Africa, with the exception of a few European countries. The probability of international spread increases if the Nigerian outbreak is not contained, particularly in Europe and the Americas. We have to stress that although we use very detailed data about traffic flows and airline scheduling, there is very scant information about the demographics of traveling individuals. The introduction of heterogeneity due to income and household type in the traveling patterns would therefore increase the accuracy of the projections. We also do not consider that some airlines have announced the suspension of the flights to and from the West African regions affected by the EVD outbreak 13 ^,^ 14. Airline companies have also announced that they will assist travelers by booking on other airline companies still operating in the region, and therefore we do not currently have the necessary data to assess the real decrease of travel to and from the region. We have explored the scenario assuming an 80% airline traffic flow reduction to and from the West African region that provided evidence of a general time-delay of the distribution characterizing the probability of case importation of about three to four weeks. This is a quite well known result backed by theoretical arguments, numerical and data evidence 15 ^,^ 16 ^,^ 17 ^,^ 18 ^,^ 19. Although this delay may be useful, it would not help much unless interventions on the ground could be put into place in the interval that were effective in stopping the growth of the EVD outbreak 20. In addition, travel restrictions may hamper the deployment of personnel and support in the region, ultimately creating a counter productive effect in the containment effort. Finally, we found that the ability to detect ebola cases during international flights can affect the risk of importation; airports that serve a high proportion of international transfers are more likely to have case importations when cases can be detected on each flight segment, while airports that are "destinations" are more likely to have importations when cases can not be detected until they reach their destination, as shown in Figure 3.

The assessment of the expected number of cases locally generated in countries experiencing the importation of the EVD assumes that outside the African region, the hospital and funeral transmissibility is negligible. Local community transmission is however allowed, as infectious individuals may not be immediately diagnosed with EVD and isolated. The distribution of outbreak sizes is also very small in countries outside the African region. However, in the modeling we do not consider the possibility of cluster importation due to household members traveling together or because of direct transmission in the plane.

## Conclusions

We show by a modeling effort informed by data available on the 2014WA EVD outbreak that the risk of international spread of the Ebola virus is still moderate for most of the countries. The current analysis however shows that if the outbreak is not contained, the probability of international spread is going to increase consistently, especially if other countries are affected and are not able to contain the epidemic. It is important to stress that the presented modeling analysis has been motivated by the need for a rapid assessment of the EVD outbreak trends and contains assumptions and approximations unavoidable with the current lack of data from the region. The results may change as more information becomes available from the EVD affected region and more refined sensitivity analysis can be implemented computationally. Furthermore, the modeling approach does not include scenarios for the identification and isolation of cases, the quarantine of contacts, and the proper precautions in hospital and funeral preparation that would be relevant in discussing optimal containment strategies. Such a modeling effort however calls for better and more detailed data not available at the moment.

## Methods

**Data**

We gathered data from the Disease Outbreaks News^1^ of the WHO. The WHO tracks new cases and deaths by date with regular reports (generally biweekly). We considered Sierra Leone, Guinea and Liberia as officially affected by the EVD epidemics. Nigeria is suffering only a small outbreak confined to Lagos and all cases are apparently linked to the index case that arrived from Liberia.

**Global Epidemic and Mobility Model**

The Global Epidemic and Mobility Model is a spatial, stochastic and individual based epidemic model, in which the world is divided into geographical regions defining a subpopulation network, where connections among subpopulations represent population traffic flows due to transportation and mobility infrastructures. The model’s technical details and the algorithms underpinning the computational implementation are extensively reported in the literature 7 ^,^ 8 ^,^ 9 ^,^ 21. By using real demographics, the model divides the world population into geographic census areas that are defined around transportation hubs and connected by mobility fluxes; this process effectively defines an infectious disease metapopulation network model 22 ^,^ 23 ^,^ 24.

The subpopulations of the model correspond to geographic census areas defined around transportation hubs obtained using a Voronoi-like tessellation of the Earth’s surface by assigning each cell of the grid to the closest transportation hub (generally airports or major urban areas) taking into account distance constraints. The population of each census area is obtained by integrating data from the high-resolution population database of the ’Gridded Population of the World’ project of the Socioeconomic Data and Application Center at Columbia University (SEDAC) 25. The model counts more than 3,300 census areas in about 220 different countries (numbers may vary by the year considered according to changes in the databases, often because of conflicts).

The mobility among subpopulations integrates the mobility by global air travel (obtained from the International Air Transport Association 26 and Official Airline Guide 27 databases) and the short-scale mobility between adjacent subpopulations, which represents the daily commuting patterns of individuals. Commuting and short-range mobility considers data from 80,000 administrative regions from countries in 5 different continents. The model also considers the modeling of mobility through different validated approaches 8 ^,^ 28. The model simulates the number of passengers traveling daily worldwide by using the real data obtained from the airline transportation databases, which contain the number of available seats on each airline connection in the world among the indexed airports. The short range commuting flows are accounted for by defining effective mechanistic subpopulation mixing 9. The minimal time scale of the model is set to one day.

The disease model within each subpopulation assumes a compartmental classification of the disease under study. The epidemic evolution is modeled using an individual dynamic where transitions are mathematically defined by chain binomial and multinomial processes 29 to preserve the discrete and stochastic nature of the processes. Each subpopulation’s disease dynamic is coupled with the other subpopulations through the mechanistically simulated travel and commuting patterns of disease carriers. The disease model used for this study is specific to the EVD and follows the compartmentalization used by Legrand *et al.* 10. The model works in discrete time steps, representing a full day, to implement computationally the air travel, the compartmental transitions (where the force of infection takes into account both the infection dynamics and the short-range movement of individuals), and the partial aggregation of the results at the desired level of geographic resolution. The model is fully stochastic and from any nominally identical initialization (initial conditions and disease model) generates an ensemble of possible epidemic evolutions for epidemic observables, such as newly generated cases, time of arrival of the infection, and number of traveling carriers.

The execution time of the stochastic simulations depends on the values of the parameters of the model. For instance, on an Intel Xeon Processor E5-2430 (6 cores @ 2.20GHz), simulating 1,000 realizations with 100 time steps in each one, for 108 different values of the parameters, took about 12 hours. A version of GLEAM for small pilot studies is publicly available (www.gleamviz.org), and is based on a client-server system.

**Disease dynamic models**

For the study of the dynamic of the disease, we use a compartmental classification of the stage of the disease. We considered two different models of increasing detail.

One parsimonious model assumes an SEIR compartmental structure where individuals are classified as it follows: susceptible individuals *S *who can acquire the infection; exposed individuals *E *that will become infectious at a rate ε**= 1*/*7 days^*−*1^; infectious individuals *I *that can transmit the disease; removed individuals *R *where the infectious individuals move at a rate *γ *= 1*/*10 days^*−*1^. The *R *compartment includes the individuals that can no longer transmit the disease because either they recovered or died. The transition probabilities are chosen for consistency with the more refined model adopted in our analysis. This model has been introduced by Legrand *et al*. 10 and individuals are classified in the following way: susceptible individuals *S*, who can acquire the disease after contact with infectious individuals, exposed individuals *E *who are infected but do not transmit the disease and are asymptomatic, infectious individuals *I *who can transmit the disease and are symptomatic, hospitalized infectious individuals *H*, dead individuals *F *that can infect through the burial ceremonies, and recovered or removed individuals *R*. In both models, individuals in the exposed state are allowed to follow usual mobility patterns and travel internationally. In Fig. 5 we show a schematic representation of the model and the transitions between compartments. In Table 1 we report in detail the transition probabilities used in this study.

From the proportion of hospitalized cases *θ*, one can obtain the hospitalization rate *θ*_1_ for the infectious compartment *I*. This can be done by assuming that *θ *corresponds to the fraction of instantaneous transitions from compartment *I *to the hospitalized compartment *H*, over all transitions originating from *I*. A similar construction is done to obtain the compartment specific death rates *δ*_1_ and *δ*_2_. For the calculation of *δ*_1_, the fatality rate for non-hospitalized infected individuals, we consider that the CFR *δ* equals the fraction of transitions from compartment *I *to *F *with respect to all transitions that do not correspond to hospitalization. The same is done for *δ*_2_, the fatality rate for hospitalized individuals, where we take *δ *equals the fraction of transitions *H *to *F *, with respect to all transitions from compartment *H*.

The expression for the basic reproductive number *R*_0_ is obtained following the method of Dieckmann & Heesterbeek 30 ^,^ 31. Legrand *et al.* 10 showed that this parameter can be written as the sum of three terms for this model: a term that accounts for the transmissions in the community, *R*_*I*_, a second term that accounts for transmissions within hospitals, *R*_*H*_, and a third that takes into account the infections from dead individuals, *R*_*F *_. As the outbreak has been developing for several months, we consider that any containment measure is already in place. Therefore any reduction in the transmissibility in each setting is already incorporated in the corresponding effective transmission rate in each compartment, *β*_*I*_, *β*_*H *_and *β*_*F *_. As shown in Ref. [10], the relationship between each compartment specific reproduction rate and transmissibility is given by

\begin{equation*}R_0 = R_I + R_H + R_F,\end{equation*}

\begin{equation*}R_I = \frac{\beta_I}{\Delta},\end{equation*}

\begin{equation*}R_H = \frac{ \theta \beta_H}{\gamma_{dh}\delta_2 + \gamma_{ih}(1-\delta_2)},\end{equation*}

\begin{equation*}R_F = \frac{\delta\beta_F}{\gamma_f},\end{equation*}

where the parameter _\begin{equation*}\Delta=\gamma_h \theta_1 + \gamma_d\delta_1 (1-\theta_1) + \gamma_i(1-\theta_1)(1-\delta_1).\end{equation*}_

**Model selection and calibration**

In view of available data, we decided to focus on the EVD transmissibility as modeled by the basic reproductive number. All the other parameters have been set as in Table 1, by using results found in the literature and showing relatively small variations across different modeling studies and different past outbreaks 10 ^,^ 11 ^,^ 12. In order to estimate the transmissibility components, we have explored *R*_0_ in the interval [0*.*7*,* **3*.*7], generating for each sampled point a statistical ensemble of 1,000 identically initialized Monte Carlo simulations of the epidemic spread at the local and global level. For the Legrand *et al*. disease model 10 we performed a latin hypercube sampling of the parameter space defined by the vector *P *= (*R*_*I*_ *, R*_*H*_ *, R* *_F_*). The transmissibility range in each setting considers values up to two times larger than those reported after intervention in Legrand at al. Simulations are initialized with the data on new cases and deaths from the official report in the four affected countries during the week of 6 July. From each statistical ensembles it is possible to estimate the likelihood function *L*(*P,* **|*x*_*i *_), where *x*_*i *_indicates the deaths data according to the WHO data during the time interval spanning from 9 July to 9 August. It shall be noted that the vector *P *, defines for each set of values a different global epidemic model through the non-parametric definition of the infection spread across different subpopulations. In other words, while the local transmission model has the same structure, the coupling among subpopulations is defined by a different non-parametric mechanistic approach. We have considered the likelihood region defined by the 1/10 relative likelihood function in defining the parameters’ range and selected models. The multimodel inference approach based on the Akaike information Criterion (AIC) is used to compare different models 32. This approach has the advantage of not assuming a best model but rather selects the likelihood of each proposed model. The reported parameters are given by the model with highest likelihood.

**Compartmental model:**
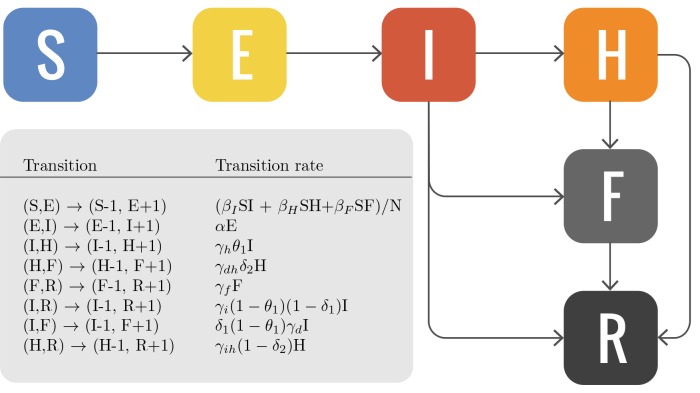
Schematic representation of the compartmental model with susceptible individuals, S; exposed individuals, E; infectious cases in the community, I; hospitalized cases, H; dead but not yet buried, F; and individuals no longer transmitting the disease, R. Model parameters are: β_I_ , transmission coefficient in the community; β_H_ , transmission coefficient at the hospital; β_F_ , transmission coefficient during funerals. θ_1_ is computed so that θ% of infectious cases are hospitalized. Compartment specific δ_1_ and δ_2_ are computed so that the overall case-fatality ratio is δ. The mean incubation period is given by α^−1^; γ_h_^−1^ is the mean duration from symptom onset to hospitalization; γ_dh_^−1^ is the mean duration from hospitalization to death; γ_i_^−1^ is the mean duration of the infectious period for survivors; γ_ih_^−1^ is the mean duration from hospitalization to end of infectiousness for survivors; and finally, γ_f_^−1^ is the mean duration from death to burial.

**Table 1. Epidemiological features used in this study.** The time spent in each compartment corresponds to the mean time reported in the different references, and used by the Legrand et al. study 10 . θ_1_ is computed in order to obtain the given proportion θ% of infectious individuals hospitalized. δ_1_ and δ_2_ are computed in order to have an overall case fatality ratio δ. δ_1_ and δ_2_ are fatality ratio parameters associated with the different compartments. For details on how to compute each one of them see Ref. [10].

Transition parameters	Value	References
Mean duration of incubation period (1*/α*)	7 days	33 ^,^ 34 ^,^ 35
Mean time from onset to hospitalization (1*/γ*_*h*_)	5 days	36
Mean time from onset to death (1*/γ*_*d*_)	9.6 days	36
Mean time from onset to end of infectiousness for survivors (1*/γ*_*i*_)	10 days	35 ^,^ 37
Mean time from death to traditional burial (1*/γ*_*f*_)	2 days	10
Proportion of cases hospitalized, *θ*	80%	36
Rate of transition from infectious to hospitalized* (θ*_1_)	0.67	
Case fatality ratio, *δ*	55%	
*δ*_1_	0.54	
*δ_2_*	0.53	
Mean time from hospitalizations to end of infectiousness for survivors (1*/γ*_*ih*_)	5 days	
Mean time from hospitalizations to death (1*/γ*_*dh*_)	4.6 days	

## Competing Interests

The authors have declared that no competing interests exist.
